# Medium Entropy Reduction and Instability in Stochastic Systems with Distributed Delay

**DOI:** 10.3390/e23060696

**Published:** 2021-05-31

**Authors:** Sarah A. M. Loos, Simon Hermann, Sabine H. L. Klapp

**Affiliations:** 1 Institut für Theoretische Physik, Technische Universität Berlin, Hardenbergstr. 36, 10623 Berlin, Germany; sabine.klapp@tu-berlin.de; 2 ICTP—The Abdus Salam International Centre for Theoretical Physics, Strada Costiera 11, 34151 Trieste, Italy; 3 Institut für Theoretische Physik, Universität Leipzig, Brüderstraße 15, 04103 Leipzig, Germany; 4 Institut für Physik, Humboldt-Universität zu Berlin, Newtonstr. 15, 12489 Berlin, Germany; simon.hermann@physik.hu-berlin.de

**Keywords:** non-Markovian dynamics, stochastic thermodynamics, time-delayed feedback control, stochastic delay differential equations, feedback cooling, non-reciprocal interactions

## Abstract

Many natural and artificial systems are subject to some sort of delay, which can be in the form of a single discrete delay or distributed over a range of times. Here, we discuss the impact of this distribution on (thermo-)dynamical properties of time-delayed stochastic systems. To this end, we study a simple classical model with white and colored noise, and focus on the class of Gamma-distributed delays which includes a variety of distinct delay distributions typical for feedback experiments and biological systems. A physical application is a colloid subject to time-delayed feedback control, which is, in principle, experimentally realizable by co-moving optical traps. We uncover several unexpected phenomena in regard to the system’s linear stability and its thermodynamic properties. First, increasing the mean delay time can destabilize or stabilize the process, depending on the distribution of the delay. Second, for all considered distributions, the heat dissipated by the controlled system (e.g., the colloidal particle) can become negative, which implies that the delay force extracts energy and entropy of the bath. As we show here, this refrigerating effect is particularly pronounced for exponential delay. For a specific non-reciprocal realization of a control device, we find that the entropic costs, measured by the total entropy production of the system plus controller, are the lowest for exponential delay. The exponential delay further yields the largest stable parameter regions. In this sense, exponential delay represents the most effective and robust type of delayed feedback.

## 1. Introduction

In addition to the omnipresent noise, many complex systems are governed by dynamical equations that involve some sort of memory or time delay. The latter may stem from delayed response in the communication between (active) constituents, retardation effects due to finite signal transmission times, or maturation times in population growth. Another common source of delay is the presence of feedback loops, which typically involve a delay due to the time lag between feeding a signal and receiving the response [1,2,3,4,5,6,7]. Feedback is a widespread mechanism in nature, encountered in various biological systems (such as gene regulatory networks [8,9,10,11,12,13,14], the cell metabolism [15,16], or the chemotactal motion of bacteria [17,18,19]); and is further implemented in numerous artificial systems (such as robots [5], autonomously driving cars [20], or quantum devices [21,22]). Recent experiments on small-scale, fluctuating systems successfully apply feedback control involving video microscopy and co-moving laser traps to create virtual potentials [23,24] or realize arbitrary interactions between colloidal particles [25,26]. Such experiments give rise to a stochastic motion with time delay of the feedback-controlled colloidal particles [2,23,24,25,26,27,28,29].

As a most simple way to mathematically describe stochastic motion with time delay, we will consider the overdamped Langevin equation

(1) $$\begin{array}{cc} {\dot{X}}_{0}\left(t\right)= & \int_{0}^{t}K\left(t-t^{\prime }\right)X_{0}\left(t^{\prime }\right)dt^{\prime }-V^{\prime }+\xi \left(t\right), \end{array}$$

with a noise term ξ, an external potential *V*, and a linear delay force, $\int_{0}^{t}K\left(t-t^{\prime }\right)X_{0}\left(t^{\prime }\right)dt^{\prime }$, where *K* defines the distribution of the delay. The delay distribution depends on the details of the underlying (feedback) mechanism. For example, in biological systems, the delay often has a rather broad distribution [11,30,31], while in control theory, a delta-distributed delay is more common, $K\left(t-t^{\prime }\right)=\delta \left(t-t^{\prime }-\tau \right)$, giving rise to a single delay time, τ. The prime physical example motivating this paper is a colloidal particle under time-delayed feedback control, where the delay distribution is, in principle, externally tunable by the involved computer.

From a practical point of view, delay is often unwanted, as it may destroy the system’s stability [32,33,34,35,36,37,38] or induce chaos [39,40]. It furthermore dramatically increases the difficulty of the mathematical description, as the combination of delay and noise yields non-Markovian stochastic processes with infinite-dimensionality [31,32,37,38,41], that are, moreover, automatically far from thermal equilibrium [42,43]. At the same time, it is known that delay can also be beneficial, for example, it can stabilize period solutions [3,44,45,46] or induce coherent oscillations [43,47], which is why delay is sometimes intentionally implemented, see [45,48,49]. Furthermore, it has recently been shown that delay may yield intriguing thermodynamic behavior. For example, a delay force acting on a colloid can induce a net heat flow from the heat bath to the colloid, i.e., a reduction of the medium entropy [43,50]. This refrigeration effect, called feedback cooling, is closely related to feedback cooling and “entropy pumping” in Markovian inertial systems with velocity-dependent feedback [51,52,53,54,55,56] (specifically, the delay force has a comparable effect as a velocity-dependent force, and both induce entropy pumping, see [43,57]). While these effects are, in principle, known, we are still only at the beginning of a full understanding of the impact of delay on stochastic dynamics. Given the omnipresence of time delay in many fluctuating systems, deepening our theoretical understanding seems indeed crucial.

In this paper, we discuss the influence of the distribution of the delay (i.e., the functional form of *K* in Equation (1)) on dynamical and thermodynamical properties in the overdamped case. In contrast to algebraically decaying delay distributions, we here focus on “short-ranged” ones that eventually decay exponentially fast in the long time limit, which are more important in the context of feedback and naturally arise in biological systems. For a recent discussion of other kernels and corresponding stability issues, see [58]. Specifically, we will perform a linear stability analysis (around local extrema of an external potential) and study how the stability boundaries shift when we change nothing but the distribution of the delay while keeping the mean delay time and the weight of the kernel constant. Gamma-distributed delays turn out to be particularly suitable to study these questions. They are versatile (including single-exponential, and delta-distributed delay, as well as distributions with a pronounced maximum of finite width), and, at the same time, are embeddable in a higher-dimensional Markovian system. We will exploit both properties in this paper. As a prominent example for thermodynamic behavior induced by delay, we will further consider how the distribution of delay influences the aforementioned “negative dissipation”. We also study the impact of colored noise. Lastly, we discuss the total entropy production and the thermodynamic efficiency for a specific realization of a feedback controller involving non-reciprocal interactions between some linear, stochastic internal degrees of freedom (which we interpret as the “memory cells”) and gives rise to a delay force with Gamma-distributed delay.

## 2. Model

We have already introduced the overdamped Langevin equation (LE) in Equation (1) that can be used, e.g., to describe the motion of a colloidal particle ($X_{0}$ being the colloidal position) under the impact of a time-delayed feedback controller. More specifically, we will consider

(2) $$\begin{array}{cc} {\dot{X}}_{0}\left(t\right)= & \int_{0}^{t}K\left(t-t^{\prime }\right)X_{0}\left(t^{\prime }\right)dt^{\prime }-V^{\prime }+\xi_{0}+\nu , \end{array}$$

which accounts for two types of noise, $\xi =\xi_{0}+\nu$. First, $\xi_{0}$ is a zero-mean Gaussian white noise with $\langle \xi_{0}\left(t\right)\xi_{0}\left(t^{\prime }\right)\rangle =2k_{B}T_{0}/\gamma_{0}\;\delta_{ij}\delta \left(t-t^{\prime }\right)$ with the Boltzmann and friction constants $k_{B}$, $\gamma_{0}$, and 〈…〉 denoting the ensemble average. This white noise stems from the surrounding heat bath at temperature $T_{0}$. Second, ν is a Gaussian colored noise which may account for a finite precision of the feedback controller [as we elaborate below, and define in Equation (6)]. The term $\int_{0}^{t}K\left(t-t^{\prime }\right)X_{0}\left(t^{\prime }\right)dt^{\prime }$ models the delay force with distributed delay. Note that the linearity in $X_{0}$ is indeed a typical assumption in the experimental realizations of feedback by co-moving laser traps [59,60].

**Figure 1:**
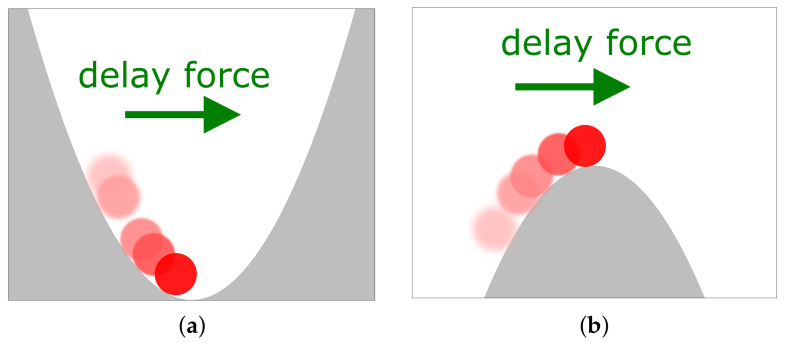
Sketch of a particle (red disk) under the influence of a force that depends on the past trajectory (indicated by lighter red disks), for different values of *a* of the external potential $V=a/2\;X_{0}^{2}$: (**a**) the particle is in a harmonic trap (a>0), (**b**) the particle is “on a parabolic mountain” (a<0). Here, the arrows indicate the force pertaining to k>0, i.e., the delay force is positive when the weighted integral of the past trajectory is negative, see Equation (1).

The external potential *V* in Equation (2) depends on the specific system at hand. An important example is $V=a_{}X_{0}^{2}/2$, giving rise to a linear force $-a_{}X_{0}$, which could represent an approximate potential gained by linearization of a more complicated *V* around a (local) extremum, or it could represent an actual parabolic potential. For a>0, the LE (2) may be used to describe a colloidal particle in an (optical) trap, while a<0 corresponds to a colloid on a “parabolic mountain”, see Figure 1b.

**Figure 2:**
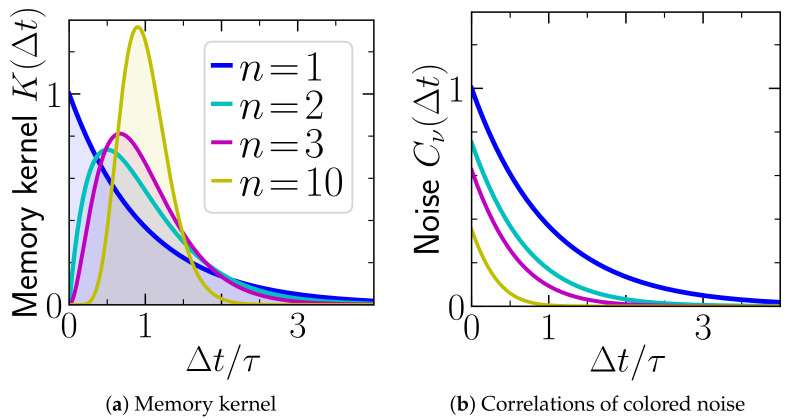
(**a**) Delay distribution given in Equation (3), and (**b**) correlations of colored noise ν given in Equation (6) for different *n*, pertaining to arbitrary values of τ, *k*, $T_{0}$, T′ and choices of *V*. The delay distributions *K* are plotted in units of |k|, while $C_{\nu }$ is plotted in units of $k^{2}/\left(\tau k_{B}T^{\prime }\right)$.

A typical feature of feedback is a time delay τ. The focus of this paper is to study the impact of different delay distributions. For this purpose, a class of Gamma-distributions defined by [61]

(3) $$\begin{array}{cc} K(\Delta t) & =k\frac{n^{n}\;\Delta t^{n-1}}{\tau^{n}\left(n-1\right)!}e^{-\frac{n\Delta t}{\tau }}, \end{array}$$

(with Δt≥0) turns out to be particularly suitable for the following reasons:

(i) By changing *n*, we can model various common types of delay with distinct characteristics, see Figure 2. For n=1, the kernel is a simple exponential decay. For n>1, K(Δt) is peaked around Δt=τ, with peaks becoming sharper upon increasing *n*. In the limit n→∞, it reaches a delta-distribution, $\lim_{n\to \infty }K\left(\Delta t\right)=k\delta \left(\Delta t-\tau \right)$ [41]. Thus the ansatz Equation (3) includes the case of a discrete (single) delay.
(ii) The weight of *K*, which defines the *feedback gain k*, is identical for all *n*: $\int_{0}^{\infty }K\left(\Delta t\right)\;d\Delta t=k$.
(iii) The *mean delay time τ* is identical for all *n*:
  (4) $$\begin{array}{c} \frac{\int_{0}^{\infty }K\left(\Delta t\right)\Delta t\;d\Delta t}{\int_{0}^{\infty }K\left(\Delta t\right)\;d\Delta t}\;=\;\tau . \end{array}$$
(iv) There exists a corresponding Markovian system that generates a memory of the form Equation (3) (as explained in the following section). Using this route allows us to obtain various analytical expressions that would be notoriously difficult to derive based on the non-Markovian LE (2) alone.

### 2.1. Markovian Representation

Alternative to the non-Markovian LE (2), one may formulate a *Markovian* equation by embedding the system in a higher-dimensional space (adding “auxiliary variables”) [41]. We note that such a Markovian representation is not unique, i.e., different Markovian models lead, after projection onto $X_{0}$, to the same non-Markovian Equation (2). Here, we will consider the (n+1)-dimensional Markovian system

(5a) $$\begin{array}{cc} \underline{\dot{X}}\; & =A\underline{X}+\underline{\xi }+\underline{\phi } \end{array}$$

(5b) $$\begin{array}{cc} A & =\left(\begin{array}{ccccc} -a_{} & 0 & 0 & ... & k\\ n/\tau & -n/\tau & 0 & ... & 0\\ 0 & n/\tau & -n/\tau & ... & 0\\ \; & \; & \vdots & \; & \\ 0 & ... & 0 & n/\tau & -n/\tau \end{array}\right) \end{array}$$

with the vector $\underline{X}={\left(X_{0}\;X_{1}\;\cdots \;X_{n}\right)}^{T}\in R^{n+1}$, and the noise vector $\underline{\xi }={\left(\xi_{0}\;\xi_{1}\;\cdots \;\xi_{n}\right)}^{T}$ containing zero-mean Gaussian white noises with $\langle \xi_{i}\left(t\right)\xi_{j>0}\left(t^{\prime }\right)\rangle =2k_{B}T^{\prime }\;\delta_{ij}\delta \left(t-t^{\prime }\right)$ and $\langle \xi_{0}\left(t\right)\xi_{0}\left(t^{\prime }\right)\rangle =2k_{B}T_{0}/\gamma_{0}\;\delta_{ij}\delta \left(t-t^{\prime }\right)$. In the case $T^{\prime }=0$, this embedding is called the “linear chain trick” [31,62]. The asymmetry of the coupling matrix A is associated with *non-reciprocal interactions* between the $X_{j}$ [57]. Further, if the external potential in Equation (2) is parabolic, $\underline{\phi }=0$, otherwise $\underline{\phi }=\left[aX_{0}-V^{\prime }\right]\;{\left(1\;0\;\cdots \;0\right)}^{T}$.

**Figure 3:**
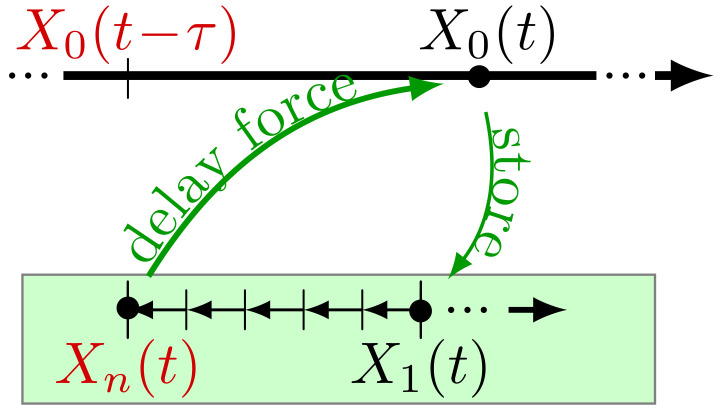
Relation between the temporal evolutions of $X_{0}$ and the $X_{j>0}$ representing the *n* “memory cells of the controller” (as further explained in Appendix A.1).

In the last part of this paper (Section 5), we will employ a physical interpretation of the Markovian representation (5). Specifically, one may interpret the variables $X_{j>0}$ as the *memory cells* of a feedback controller, which has a shift register architecture. This idea is illustrated in Figure 3, and further elucidated in Appendix A.1. The noise terms $\xi_{j>0}$ then give the controller degrees of freedom a finite temperature $T^{\prime }$. Alternatively, one may interpret the terms $\xi_{j>0}$ as errors of the memory device, in which it seems appropriate to define their amplitudes as *n*-dependent quantities, see the discussion in Section 2.4.

To return from Equations (2)–(5) one can employ a projection [41,63], as we derive in Appendix A. This gives rise in Equation (2) to the delay distribution (Equation (3)) and the Gaussian colored noise ν with noise correlations (for Δt≥0)

(6) $$C_{\nu }\left(\Delta t\right)=\langle \nu \left(t\right)\nu \left(t+\Delta t\right)\rangle =\frac{k_{B}T^{\prime }}{k^{-2}}\sum_{p=0}^{n-1}\sum_{l=0}^{p}\frac{2^{l-2p}\left(2p-l\right)!}{p!(p-l)!l!}\frac{e^{-n\Delta t/\tau }\Delta t^{l}}{{\left(\tau /n\right)}^{l-1}}.$$

We emphasize that the dynamics of $X_{0}$ is *identical* in both representations (2) and (5).

### 2.2. Colored Noise

Let us take a closer look at the colored noise (Equation (6)). It is zero for $T^{\prime }\equiv 0$ (corresponding to a deterministic controller), and otherwise positive. Interestingly and contrary to the delay distribution *K*, which dramatically changes between n=1 and n=2, the correlations of the colored noise (Equation (6)) remain very similar for all *n*. They decay monotonically in all cases (see Figure 2). Indeed, as we have explicitly shown in [57], the system with colored noise (Equation (6)) and delay distribution (Equation (3)) never fulfills the fluctuation-dissipation relation (FDR) of second kind, indicating the inherent non-equilibrium nature of the non-Markovian process $X_{0}$ (Equation (2)). An exception is the case n=1 with

(7) $$\begin{array}{c} k\tau =T_{0}/T^{\prime }, \end{array}$$

where FDR is fulfilled. As we have discussed in [57], a way to understand the broken FDR are the involved non-reciprocal, interactions in the corresponding Markovian representation (Equation (5)). We have further shown in [57] that the detailed balance (DB) condition is as well only fulfilled for n=1 and condition (Equation (7)).

### 2.3. Limit of Infinitely Large System

In many physical and biological systems, a delay distribution with a non-zero width is the more realistic scenario [61,64]. For example, in the context of feedback, the non-zero width may come from a finite precision and resolution of the control device. In our interpretation of $X_{j>0}$, this corresponds to a finite number *n*, i.e., the controller has a finite memory capacity. However, in theoretical studies, the delay is often assumed to be discrete, i.e., infinitely sharp. In our approach, this corresponds to the limit n→∞, where K(Δt)→δ(Δt−τ) [41].

In this limit, we find a surprising result for the colored noise, that is, the noise correlation entirely *vanishes*

(8) $$\lim_{n\to \infty }C_{\nu }\left(\Delta t\right)=0$$

(irrespective of the value of $T^{\prime }$), implying that the colored noise ν itself vanishes. Hence, Equation (2) becomes a delay equation with white noise, reading

(9) $${\dot{X}}_{0}\left(t\right)=-a_{}X_{0}+k\;X_{0}\left(t-\tau \right)+\xi_{0}.$$

**Proof.**  We perform the limit of n→∞ of the noise correlation given in Equation (6), which read for Δt≥0

(10) $$\frac{C_{\nu }\left(\Delta t\right)}{k_{B}T^{\prime }k^{2}}=\sum_{p=0}^{n-1}\sum_{l=0}^{p}\frac{n^{l-1}\left(2p-l\right)!}{\tau^{l-1}2^{2p-l}p!\left(p-l\right)!l!}e^{-n\Delta t/\tau }\Delta t^{l}.$$

To this end, we calculate the weight of Equation (10), i.e., $\Psi =\int_{0}^{\infty }C_{\nu }/\left(k_{B}T^{\prime }k^{2}\right)d\left(\Delta t\right)$, yielding

(11) $$\Psi =\sum_{p=0}^{n-1}\sum_{l=0}^{p}\frac{2^{l-2p}\left(2p-l\right)!}{{\left(\tau /n\right)}^{l-1}p!\left(p-l\right)!l!}\;\frac{\tau^{l+1}}{n^{l+1}}\int_{0}^{\infty }e^{-u}u^{l}\;du$$

This can further be simplified to

(12) $$\Psi =\frac{\tau^{2}}{n^{2}}\sum_{p=0}^{n-1}\sum_{l=0}^{p}\frac{1}{2^{2p-l}}\frac{(2p-l)!}{p!(p-l)!l!}\;l!=\frac{\tau^{2}}{n^{2}}\sum_{p=0}^{n-1}\sum_{l=0}^{p}\frac{(2p-l)!}{p!(p-l)!}{\left[\frac{1}{2}\right]}^{2p-l}.$$

Now we use the binomial theorem to further simplify this expression, and find

(13) Ψ=τ2n2∑m=1n∑r=0ppr12r12p−r=τ2n2∑m=1n12+122(n−m)=τ2n2∑m=1n1=τ2n.

Hence, the weight *vanishes* as n→∞. Since $C_{\nu }$ is obviously a non-negative function of Δt, this readily implies (8). □

### 2.4. Alternative Choice of $T^{\prime }$

So far, we have considered an *n*-independent temperature of the auxiliary variables, $T^{\prime }$, which seems a reasonable assumption when $T^{\prime }$ describes the temperature of the “controller degrees of freedom” $X_{j>0}$. Alternatively, one may scale the temperatures of the auxiliary heat baths with *n*, specifically, $T^{\prime }=nT^{\prime \prime }$ in Equation (5). In Appendix A.1, we provide a reasoning why this could be a better choice when the noises $\xi_{j>0}$ are interpreted as errors of the controller. Using the same reasoning as before one may show that, in the alternative choice, the colored noise in Equation (2) has a weight Ψ which is *independent* of *n* [contrary to the result Equation (13)]. The corresponding equation for n→∞, i.e., Equation (9), then contains a white noise at temperature $T_{0}+T^{\prime }{\left(k\tau \right)}^{2}$. Thus, this choice of $T^{\prime }$ results in controller errors whose effect does not vanish as n→∞, which seems indeed more plausible than the standard choice, where the controller errors vanish in this limit. We note that all of the following results also apply to this alternative choice just by setting $T^{\prime }=nT^{\prime \prime }$.

## 3. Stability for Different *n*

Let us now turn to the dynamics of $X_{0}$. A prominent property of systems with delay are delay-induced oscillations (which can generally not occur in first order ODEs, but arise in first order DDEs [33]). In linear systems, they are either damped, such that a stable steady state is reached in the limit t→∞, or their amplitude increases with time yielding instability. Here we explore whether, for a given $a_{}$, *k*, τ and *n*, the system reaches a stable steady state (for more mathematical discussions of noise-free systems with Gamma-distributed delay, see [65,66]). For a stochastic system (2) with a general potential *V*, the following analysis determines the linear stability of the fixed points. As an illustration, imagine the task to stabilize a particle on a parabolic mountain (when $a_{}<0$) by means of a delayed feedback force (Figure 1). As we now show, the system’s stability significantly depends on the distribution of the delay, even if the feedback gain and mean delay time stay the same.

Determining the stability of the noisy process $X_{0}$ amounts to checking whether the moments stay finite or diverge as t→∞. In the here considered examples, the steady state probability density is Gaussian [32,67] (with zero mean due to the system’s symmetry). Thus, we need to consider the second moment, $\langle X_{0}^{2}\left(t\right)\rangle$. A calculation of the latter for different *n* is provided in Appendix B. For n>1, this yields cumbersome integral expressions. Interestingly, we found that the stability boundaries, however, do not depend on the temperatures $T^{\prime }$ and $T_{0}$. We conclude that the type of noise or the presence of noise at all does not have any impact on the linear stability of the system. This includes, in particular, the colored noise and the related additional memory.

Owing to these insights, we here discuss a simpler route to check the stability based on the noise-free case ($T_{0}=T^{\prime }\equiv 0$), where (5) reduces to

(14) $$\underline{\dot{X}}=A\underline{X}.$$

This linear matrix equation has solutions of the form $\underline{X}=\underline{X}\left(0\right)e^{At}$, such that the stability boundaries can be simply determined by calculating the real part of the largest eigenvalue of the coupling matrix A, called λ. If λ>0, the system is unstable, while it is stable if λ<0.

Due to the sparseness of A, this strategy allows to determine the stability boundaries up to very large values of *n*. However, it is still limited to finite *n*. To determine to stability in the limit n→∞, we consider directly the noise-free delay Equation (9), that is

(15) $${\dot{X}}_{0}=-a_{}X_{0}\left(t\right)+kX_{0}\left(t-\tau \right).$$

This equation can also be solved by an exponential ansatz $X_{0}\left(t\right)=X_{0}\left(0\right)e^{-\hat{\lambda }t}$, yielding the transcendental equation [68,69]

(16) $$\hat{\lambda }-a_{}=-ke^{\hat{\lambda }\tau }.$$

In the trivial cases k=0 and τ=0, the solutions are $\hat{\lambda }=a_{}$ and $\hat{\lambda }=a_{}-k$, respectively, immediately giving some stability boundaries. Further, if $k=a_{}$, a solution is $\hat{\lambda }=0$. In general, the solutions of Equation (16) read [68,69]

(17) $${\hat{\lambda }}_{m}=-\frac{1}{\tau }\left[W_{m}\left(ke^{a_{}\tau }\tau \right)-a_{}\tau \right],$$

with the infinitely many branches of the Lambert-W function $W_{m}$, m∈Z. The branch m=0 has the highest real part. Thus, the stability of the DDE changes at ${\hat{\lambda }}_{m}=0$.

**Figure 4:**
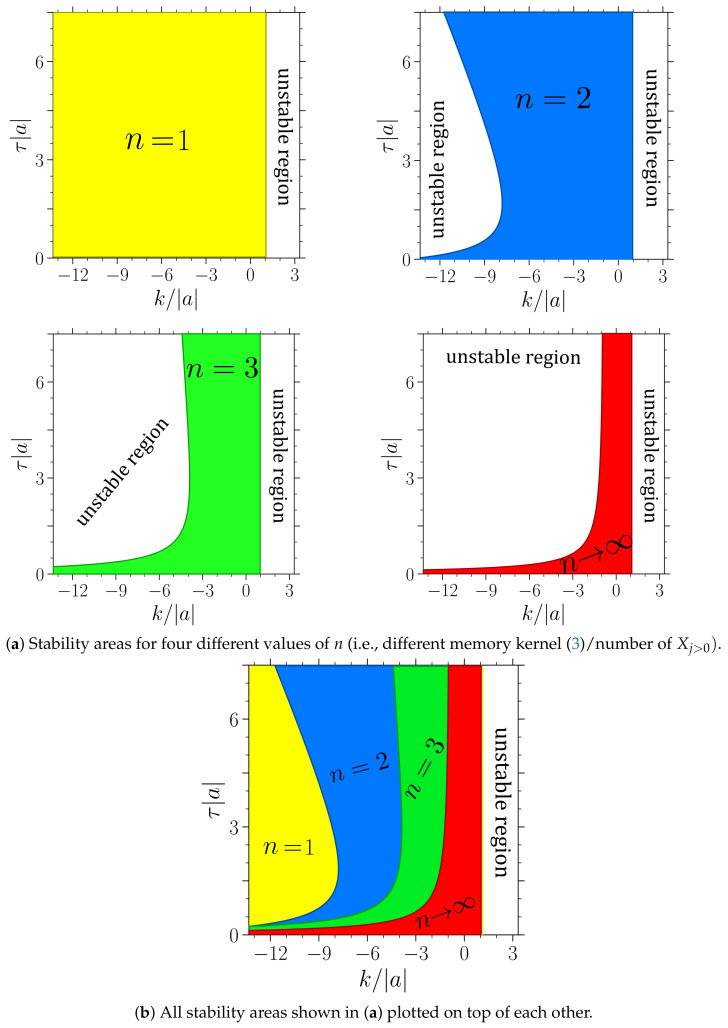
Stable regions of the stochastic process with $a_{}>0$ (“particle in harmonic trap”, see Figure 1a), in a plane spanned by the feedback gain *k* given in units of |a| and delay time τ, given in units of 1/|a|. White areas denote unstable behavior. Four panels of (**a**): Stability boundaries for the cases n=1 (yellow), n=2 (blue), n=3 (green), and n→∞ (red). (**b**): All stability areas from panels (**a**) plotted on top of each other. The size of the stability areas decreases with *n*. For example, in the red area, all systems up to n→∞ are stable. The regions exceed the shown parameter range and continue towards smaller *k* and larger τ.

**Figure 5:**
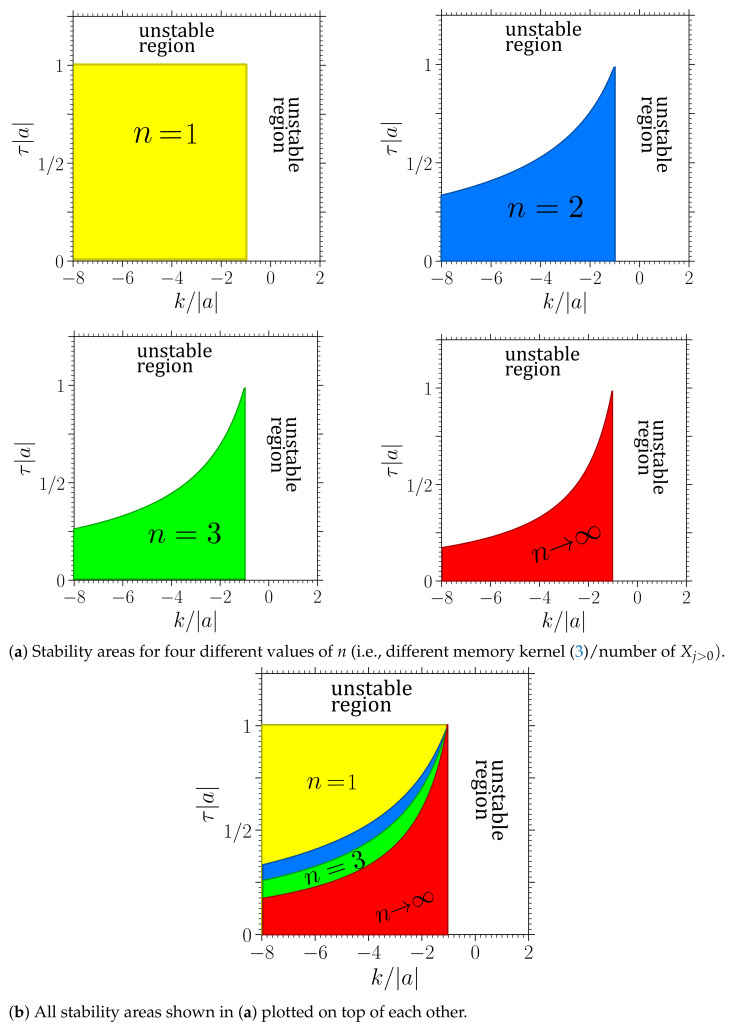
Stable regions of the stochastic process with $a_{}<0$ (“particle on the parabolic mountain”, see Figure 1b). Color code as in Figure 4. Note that the regions exceed the shown parameter range and continue towards smaller *k*.

Figure 4 and Figure 5 show the stability boundaries for various *n* of the deterministic system with $a_{}>0$ and $a_{}<0$, respectively, in the plane spanned by the feedback gain *k* and mean delay time τ (given in units of |a| and 1/|a|, respectively). Note that the figures hold for arbitrary values of $a_{}>0$ and $a_{}<0$, respectively. The parameter |a| can be scaled out from the noise-free Langevin equation upon rescaling the time by t→t/|a|. The system in Figure 4 (where $a_{}>0$) corresponds to a colloidal particle in a harmonic trap, which is as expected stable in the absence of delay force (k=0 or τ=0). In contrast, the system displayed in Figure 5 (where $a_{}<0$) without delay force corresponds to a colloidal particle in a reversed trap, i.e., on a “parabolic mountain”, which is not stable, consistent with our expectation. However, in the presence of delayed feedback, stable and unstable regions emerge for both signs of *a* and for all values of *n*.

Figure 4 and Figure 5 reveal that there is a critical *k* value of $k^{*}=a_{}$ above which the system is generally unstable for all *n*. To get an intuitive understanding for this critical value, we assume for a moment that the particle would for *all* past times $t^{\prime }<t$ stand still, i.e., $X_{0}\left(t^{\prime }\right)\equiv X_{0}\left(t\right)$. Under this assumption $\int_{0}^{t}K\left(t-t^{\prime }\right)X_{0}\left(t^{\prime }\right)dt^{\prime }=kX_{0}\left(t\right),\forall n$ because $\int_{0}^{t}K\left(\Delta t\right)\;d\Delta t=k$, such that Equation (2) reduces to

(18) $${\dot{X}}_{0}\left(t\right)=\left(k-a_{}\right)\;X_{0}\left(t\right),$$

irrespective of the value of *n* (including finite *n* and n→∞), and irrespective of the values of *k*, *a* and τ≥0. Now, it becomes clear that the system is generally unstable if $k>a_{}$, and that $k^{*}=a_{}$ is a critical value (for all *n*).

We now focus on the regions *below* $k^{*}$, where the system with τ=0 is stable, but may be destabilized by the delay. In this region, we make several interesting observations.

Most prominently, the system with exponentially distributed delay (n=1) has the *largest* stable area for positive and negative $a_{}$. Further, the stability region of every $n=\tilde{n}$, includes the whole $n=\tilde{n}+1$ region, while the reverse is never found. This means, the system generally becomes less stable as the delay distribution gets more localized around the delay time τ. This trend (which has also been found in the mathematical analysis of corresponding deterministic systems [65]) is very robust, and also holds for higher *n* (e.g., n=4,5,6) and other $a_{}$. To achieve stability, it appears to be beneficial to take into account a “larger fraction of the past”. Thus, if the aim of a controller was to stabilize $X_{0}$ at a given location, the feedback with exponential delay would be the most effective and robust one.

Another remarkable aspect is the behavior for large mean delay times, τ. Only if $a_{}<0$ (“particle on the parabolic mountain”), increasing the mean delay time seems to generally destabilize the system. Then there even exists a maximal τ value of $\tau^{*}=-1/a_{}$, where the system is unstable for all *n* and *k*, see Figure 5. For $a_{}>0$ (“particle in the trap”), the behavior is very different. Here, an increased mean delay time can be both, disadvantageous or advantageous for stability (depending on *n* and *k*). Interestingly, for *n* values 1<n<∞, we observe a *reentrant* behavior: by increasing the mean delay time, the linear stability is destroyed, but by further increasing τ, the system becomes stable again. We suspect that this behavior is a consequence of an interplay with an internal timescale, specifically, the relaxation time within the parabolic potential well, which is of order $∼1/|a_{}|$. (Note that this estimate stems from solving just the conservative part on the right hand side of the LE ${\dot{X}}_{0}=-aX_{0}$. Strictly speaking, in the presence of the delay force, all timescales may be shifted, including the relaxation times.). If the mean delay time matches this internal timescale, the delay-induced oscillations might be resonantly enhanced, destroying the stability. However, this reentrant behavior is not observed for n→∞ and n=1.

## 4. Delay-Induced Heat Flow

**Figure 6:**
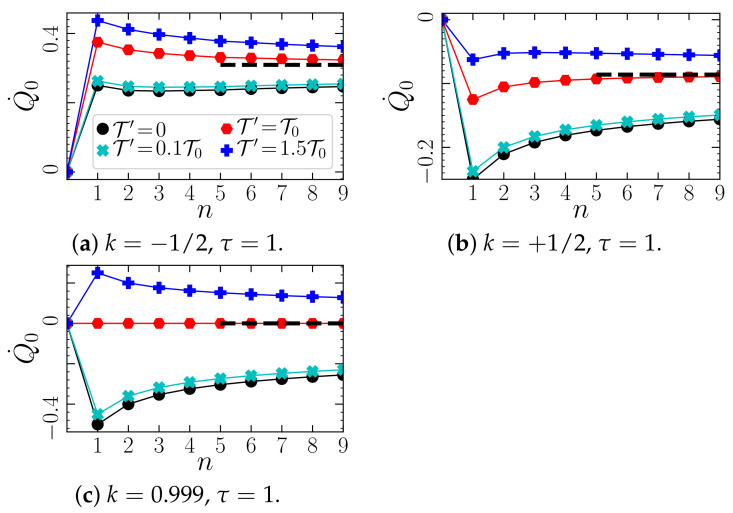
Heat flow (Equation (21)) vs. *n* in the system with a=1 (corresponding to Figure 1a and Figure 4, for different temperature ratios $T^{\prime }/T_{0}$ (different colors), and different feedback gains: (**a**) k=−1/2, (**b**) k=1/2, and (**c**) k=0.999. The parameter values in (**c**) lie for all *n* just next to the right stability boundary, which is given by $k^{*}=a=1$ (and arbitrary τ). Further, the system with n=1 in (**c**) fulfills approximately FDR, see Equation (7). The dashed lines show the solution for the discrete delay case Equation (A16), which the system expectantly approaches in the limit n→∞ (as follows from the analytical reasoning in Section 2.3). $k_{B}$ is set to unity.

Let us now turn to a thermodynamic consideration of the systems. To this end, we utilize and extend results from Ref. [57], where we have explored the connection between coupling topology A and the thermodynamic properties for generic linear systems of the type (5). We note that more general coupling topologies also allow to model other physical systems, as active particles [57]. Here we will focus on the topology Equation (5b), which yields the Gamma-distributed delay distributions. We will further give two special cases our main attention: the case of isothermal conditions, i.e., system and controller have the same temperature $T^{\prime }=T_{0}$ (red symbols in the Figure 6 and the figures below), and the case of a “perfect” (noise-less) controller, i.e., $T^{\prime }=0$ (black symbols in Figure 6).

In steady state, the mean Shannon entropy of the system, i.e., $\langle -k_{B}\ln\rho_{0}\left(x\right)\rangle$ [70] is, per se, conserved (because $\partial_{t}\rho_{0}=0$). However, nonequilibrium steady states are typically characterized by an entropy flow between system and its bath associated with heat flow. We therefore start by studying the magnitude and sign of the steady-state mean heat flow ${\dot{Q}}_{0}$ between $X_{0}$ and its bath induced by the delay force, which is proportional to the medium entropy production rate ${\dot{S}}_{m}={\dot{Q}}_{0}/T_{0}$. According to the framework by Sekimoto [71], the stochastic heat $\delta q_{0}$ flowing from $X_{0}$ to its bath, i.e., the *dissipation* of $X_{0}$ per infinitesimal time step dt is given by [71]

(19) $$\delta q_{0}/dt=\left[{\dot{X}}_{0}-\xi_{0}\right]\circ {\dot{X}}_{0}.$$

Note that we define the heat such that $\delta q_{0}>0$ indicates energy transfer from the particle to the bath (different from [71]). The heat flux (19) is independent of the question whether the non-Markovian (2) or the Markovian description (5) is employed. We will thus exploit the latter, allowing us to handle the system analytically in the case of linear forces. We are particularly interested in the *mean* heat production or dissipation rate, and find from Equations (5) and (19)

(20) $${\dot{Q}}_{0}:=\langle \delta q_{0}/dt\rangle \overset{\left(5\right)}{=}\langle \left[-a_{}X_{0}+\;kX_{n}\right]\circ {\dot{X}}_{0}\rangle .$$

Further, in the steady state, $\langle X_{j}{\dot{X}}_{j}\rangle$-correlations vanish, since $2\langle X_{i}\dot{X_{i}}\rangle =d\langle X_{i}^{2}\rangle /dt=0$, such that the mean heat production can be expressed based on the positional correlations only

(21) $${\dot{Q}}_{0}=k^{2}\langle X_{n}^{2}\rangle -k\;a_{}\langle X_{0}X_{n}\rangle .$$

From Equation (21), analytical expressions for ${\dot{Q}}_{0}$ can be derived for any *n*, as outlined in Appendix B. Noteworthy, the steady-state heat production rate equals the work applied to the system by the external delay force [70,71]. Using the closed-form expressions $\langle X_{1}^{2}\rangle$, $\langle X_{0}X_{1}\rangle$ from [57,72], one finds from Equation (21) for the case n=1

(22) $$\frac{{\dot{Q}}_{0}}{k_{B}}=\frac{T_{0}\left(k^{2}/\tau^{2}\right)-T^{\prime }k^{3}/\tau +T^{\prime }ak^{2}/\tau -T_{0}k\;a_{}/\tau^{2}}{\left(a+1/\tau )(a/\tau -k/\tau \right)}.$$

From this expression, we see that the heat flow rate diverges at both previously determined stability boundaries $k^{*}=a$, or $\tau^{*}=-1/a$. This shows that, in the unstable regions, the transient dynamics is accompanied by a medium entropy production rate that is unboundedly growing with time. Analogous behavior at the stability boundaries is also found for n>1. Within the stable regions, we can further simplify Equation (22) and find

(23) $${\dot{Q}}_{0}=k_{B}\frac{k^{2}T^{\prime }-\left(k/\tau \right)T_{0}}{a_{}+1/\tau }.$$

From Equation (23), one immediately sees that for n=1 the heat flow between $X_{0}$ and its bath vanishes when the condition for the FDR (7) is fulfilled, consistent with the expectation that the system then reaches equilibrium.

Let us now discuss the general behavior for different *n*, i.e., different delay distributions. Figure 6 shows analytical results for the mean heat rate as functions of *n*, for three different values of the feedback gain *k*. We here mainly focus on the case a>0, such that we can consider both negative and *positive k* (in stable regions). Note that, in experiments with optical traps, usually the case k>0 is of more practical relevance. First, we notice that the net heat flow of $X_{0}$ is generally nonzero, even if the temperature of $X_{0}$ and all $X_{j>0}$ is the same (red symbols), showing that the systems considered here are out of equilibrium. An exception is the case k=1/τ and $T_{0}=T^{\prime }$, where the medium entropy production rate vanishes. For this special case, the FDR is approximately fulfilled for n=1 (but violated for n>1 [57]). Further, we find that the magnitude of the induced heat flow is generally maximal for n=1, and decreases for larger *n*. Thus, the exponentially decaying delay generally yields the highest entropy flow between system and bath.

Another observation from Figure 6 is that for k>0, the heat flow from the colloid to the bath is negative (${\dot{Q}}_{0}<0$), corresponding to medium entropy *reduction*. This means that we have a steady state in which the delay force consistently extracts energy from the heat bath, i.e., feedback cooling. A Markovian external force could not have this impact on the stochastic system (as follows from the second law of thermodynamics). Importantly, this phenomenon also occurs when the “controller” $X_{j>0}$ has the same temperature as the colloid, i.e., isothermal conditions (Figure 6c), or even if the controller is hotter (then the heat flow is “reversed”) (Figure 6b). From Equation (23) follows for the case n=1 the following condition to find feedback cooling and medium entropy reduction

(24) $$0<k\tau <T_{0}/T^{\prime }.$$

On the level of the Markovian “supersystem” (colloid plus controller), the negative heat flow can be explained by the involved non-reciprocal couplings [57]. Using the non-Markovian model alone, the explanation is more subtle. An extensive discussion of the origin of the heat flow, which is connected to non-trivial information flow from the colloidal system to the controller appearing in the generalized second law for the controlled system, is provided in [57]. As an example for a nonlinear system, we have further numerically explored the heat flow in a bistable potential $V\propto (x_{0}^{4}-4x_{0})$; and again found regimes where a delay force induces a negative heat flow (for all values of *n*).

**Figure 7:**
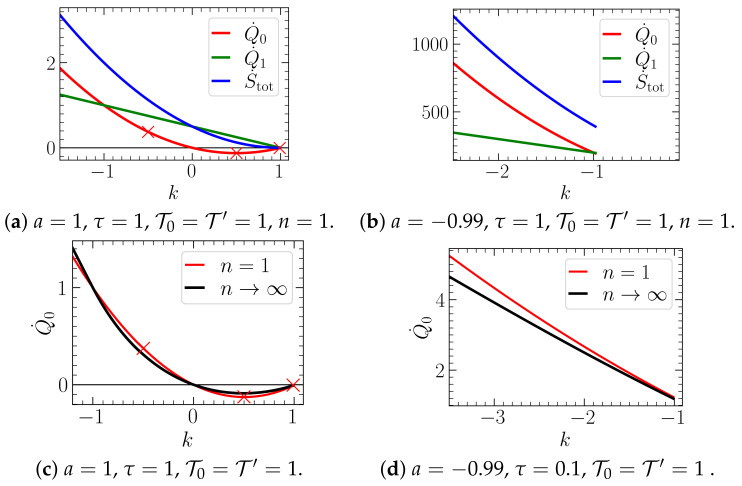
(**a**,**b**) Heat flow of $X_{0}$ (Equation (23)), total entropy production rate (Equation (29)) and heat flow of $X_{1}$ (Equation (28)) for the system with n=1 and different values of *a*: (**a**) a=1 (“particle in a trap”) and (**b**) a=−0.99 (“particle on parabolic mountain”). (**c**,**d**) Heat flow of $X_{0}$ (Equations (23) and (A16)) for the cases n=1 (i.e., exponential memory kernel) and n→∞ (delta-distributed memory kernel) in the system with (**a**) a=1 and (**b**) a=−0.99. In (**a**,**c**), the parameter settings correspond to the parameters in Figure 6. Specifically, the red crosses mark the values of *k* considered in Figure 6 panel (**a**–**c**), respectively. In all panels, the parameter values approach from the left a stability boundary given by $k^{*}=a$. Further, the parameters in (**b**) lie very close to the stability boundary $\tau^{*}=-1/a_{}$. $k_{B}$ is set to unity. For a discussion of the dependency of the heat flow on τ, which is a bit more subtle, we would like to refer the reader to [43].

In Figure 7, we display the heat flow ${\dot{Q}}_{0}$ as a function of *k*, for positive and negative *a*, for the systems with n=1 and n→∞. We observe that feedback cooling is only possible for a>0. The reason is that only in this case, stable parameter regimes with k>0 exist, for all *n* (see Section 3). Further, we observe that the heat flow ${\dot{Q}}_{0}$ displays very similar behavior in the systems with n=1 and n→∞ for all considered values of *k* and τ.

Concerning the connection to the stability analysis presented in the previous section, we note that the parameter combination considered in Figure 6c corresponds for all *n* almost to the stability boundary $k^{*}=a=1$ (see Figure 4 for a comparable case); i.e., slight increase of *k* would result in an unstable dynamics. In that case, the heat rate diverges due to the divergence of correlations entering Equation (21). However, when approaching this boundary from the left, the heat rate does not exhibit any noticeable difference compared to the parameter settings in the middle of the stability region. On the contrary, if the stability boundary at $\tau^{*}=-1/a_{}$ is approached, we notice very high values of the heat rate, see Figure 7b for an example.

Let us now consider the limit n→∞. Recall that in this limit the colored noise ν vanishes, while the delay distribution becomes a delta-distribution around τ [see Equation (9)]. We have intensely studied the heat flow in such a system in [43], for linear and nonlinear systems. The data in Figure 6 indeed seems to converge to the result from [43] (given in Appendix D) as *n* increases (as follows from the reasoning in Section 2.3). We expect the convergence to be more obvious in a regime of *n* larger than the regime that we can study on the bases of our analytical solutions. Preliminary numerical simulation results confirm this expectation, but we have not studied this question rigorously yet, and feel that it is beyond the scope of this manuscript. Remarkably, the approach is fastest for isothermal conditions $T_{0}=T^{\prime }$. In fact, for this specific case, the heat flow behaves very similar to the the heat flow in the limit n→inf for all *k* values, see Figure 7c. To us, the reason for this fastest convergence for $T_{0}=T^{\prime }$ is not clear and represents an interesting perspective of future research.

Finally, we consider the impact of the colored noise ν, which in our model stems from the noise terms $\xi_{j}$ in Equation (5) and, thus, from a finite controller temperature, or from controller errors (depending on the interpretation). Figure 6 shows that the heat flow of $X_{0}$ generally increases with the magnitude of the colored noise, $T^{\prime }$. Thus, this additional noise generally yields an additional *positive* contribution to the heat dissipated by the colloid into its heat bath (irrespective of the sign of the heat flow induced by the delay force). This trend persists (not shown here) when we employ the alternative interpretation of the noises associated to $X_{j>0}$ (see Section 2.4), i.e., when we set $T^{\prime }=nT\prime \prime$.

Taken together, we conclude that, if the aim of the controller was to cool down the surrounding fluid (like a microscopic refrigerator), the feedback with exponential delay (n=1) and without controller errors ($T^{\prime }=0$) would be the most effective one.

## 5. The Total Entropy Production

Recent literature for (underdamped) systems with *discrete* delay [42,50,73] has pointed out strategies (and related difficulties) in calculating measures of irreversibility based on the non-Markovian dynamics of $X_{0}$ alone. Here, we rather focus on the total entropy production of the supersystem consisting of particle plus controller, assuming that all variables in Equation (5) are physical. In other words, we from now on interpret the $X_{j>0}$ as the internal degrees of freedom of the controller, which are non-reciprocally coupled among each other and generate a time-delayed feedback force on the particle $X_{0}$ (as illustrated in Figure 3). A main goal of the subsequent analysis is to explore how the total entropy production depends on *n* and thus, on the sharpness of the delay distribution.

To calculate the dissipated energy of the total supersystem (particle plus controller), we employ the standard formalism from stochastic thermodynamics [70,74]. The total EP along a fluctuating trajectory $\underline{X}$$=\{X_{0}\left(t^{\prime }\right),..,X_{n}\left(t^{\prime }\right)\},$$t^{\prime }\in \left[t_{s},t_{f}\right]$ is given by

(25) $$\frac{\Delta s_{\mathrm{tot}}\left[\underline{X}\right]}{k_{B}}=\ln\frac{P[\underline{X}]}{\hat{P}\left[\hat{\underline{X}}\right]}=\frac{\Delta s_{\mathrm{sh}}}{k_{B}}+\ln\frac{P[\underline{X}|{\underline{x}}_{s}]}{\hat{P}\left[\hat{\underline{X}}|{\underline{x}}_{f}\right]},$$

involving the multivariate joint Shannon entropy $s_{\mathrm{sh}}=-k_{B}\ln\left[\rho_{n+1}\left(\underline{x}\right)\right]$ of the (n+1)-point joint probability density function (pdf) $\rho_{n+1}$, and the path probabilities P and $\hat{P}$ for forward and backward process. P conditioned on the starting point $\underline{X}\left(t_{s}\right)={\underline{x}}_{s}$, is essentially the exponential of the Onsager–Machlup action, stated in Appendix C. We assume that $X_{j>0}$ are even under time-reversal, like positions, which is more suitable when $X_{j>0}$ model memory cells (odd parity would imply information storage in particle velocities). This assumption implies

(26) $$\ln\frac{P[\underline{X}|{\underline{x}}_{s}]}{\hat{P}\left[\hat{\underline{X}}|{\underline{x}}_{f}\right]}=\int_{t_{s}}^{t_{f}}\frac{\delta q_{0}}{T_{0}}+\sum_{j=1}^{n}\int_{t_{s}}^{t_{f}}\frac{n/\tau \left(X_{j-1}-X_{j}\right)\circ dX_{j}}{T^{\prime }},$$

where $\delta q_{0}$ can be identified as the heat flow from $X_{0}$ to its bath (20). In the steady state, $\langle \Delta s_{\mathrm{sh}}\rangle :=\Delta S_{\mathrm{sh}}\equiv 0$. Applying the analogous steps as between Equations (20) and (21), we find that the ensemble average of the EP (25) reads

(27) $$\begin{array}{cc} {\dot{S}}_{\mathrm{tot}}= & \frac{{\dot{Q}}_{0}}{T_{0}}+\sum_{j=1}^{n}\frac{{\dot{Q}}_{j}}{T^{\prime }}\geq 0, \end{array}$$

(28) $$\begin{array}{cc} {\dot{Q}}_{j}= & \frac{\langle X_{j-1}^{2}\rangle -\langle X_{j-1}X_{j}\rangle }{{\left(\tau /n\right)}^{2}}. \end{array}$$

with ${\dot{Q}}_{0}$ from (21). The nonnegativity of ${\dot{S}}_{\mathrm{tot}}$ follows by construction from Equation (25), see [70]. Equation (27) yields analytical expressions for the total EP for any *n*, see Appendix B. For n=1 (which was also discussed in [72]), this yields a particularly simple closed-form expression, which diverges in the unstable regions ($k\geq k^{*}$, or $\tau \geq \tau^{*}$), and in the stable region reads

(29) $${\dot{S}}_{\mathrm{tot}}=\frac{k_{B}}{T_{0}T^{\prime }}\frac{{\left(\tau T_{0}-kT^{\prime }\right)}^{2}}{a_{}+1/\tau }.$$

The mean EP (27) has a contribution stemming from the dissipation of $X_{0}$ to its bath, ${\dot{Q}}_{0}$, and a contribution from the $X_{j>0}$, i.e., the dissipation of the additional d.o.f., ${\dot{Q}}_{j}$. This second contribution can be considered as the “entropic cost” of the memory [42,50,73] (where the “memory” arises in the form of colored noise and distributed delay). It should be emphasized, however, that memory is not unequivocally connected to EP. For example, the n=1 case with parameters such that FDR is fulfilled, also has memory in the process $X_{0}$, but no EP, see Equation (29). From Equation (29), one immediately sees that the EP vanishes if, *and only if*, the condition for the FDR (7) is fulfilled. In all other cases, the process (5) has entropic cost.

Note that the total EP is also positive for isothermal conditions $T^{\prime }=T_{0}$ (red symbols) where no temperature gradients are present in the system. The EP is closely connected with the non-reciprocal couplings between the $X_{j}$, which are non-conservative interactions [57]. We further note that the total EP is positive even in the cases where we detected the reversed heat flow, showing that the second law of thermodynamics holds for the supersystem (“the colloidal system plus the controller”), as expected. In Figure 7, we further show for the case n=1, as a function of *k*, both heat flows (${\dot{Q}}_{0}$ and ${\dot{Q}}_{1}$) and the total entropy production, which grows like $∼k^{2}$ (as also can be seen from Equation (29)).

**Figure 8:**
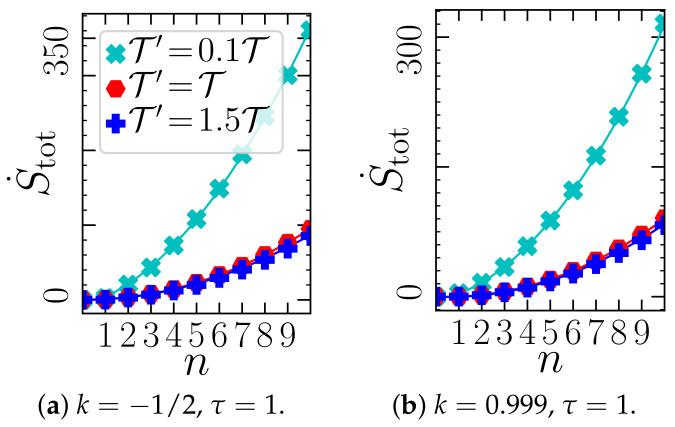
Total EP (Equation (27)) vs. *n* (giving the number of $X_{j>0}$) for a system with a=1(corresponding to Figure 1a, Figure 4 and Figure 6), at different values of feedback gain: (**a**) k=−1/2 and (**b**) k=0.999. The analytical results are complemented by quadratic fits ${\dot{S}}_{\mathrm{tot}}∼n^{2}$ (solid lines). The parameter values in (**b**) lie just next to the right stability boundary $k^{*}=a=1$ (for all *n*). All other parameters, and $k_{B}$ are set to unity.

**Figure 9:**
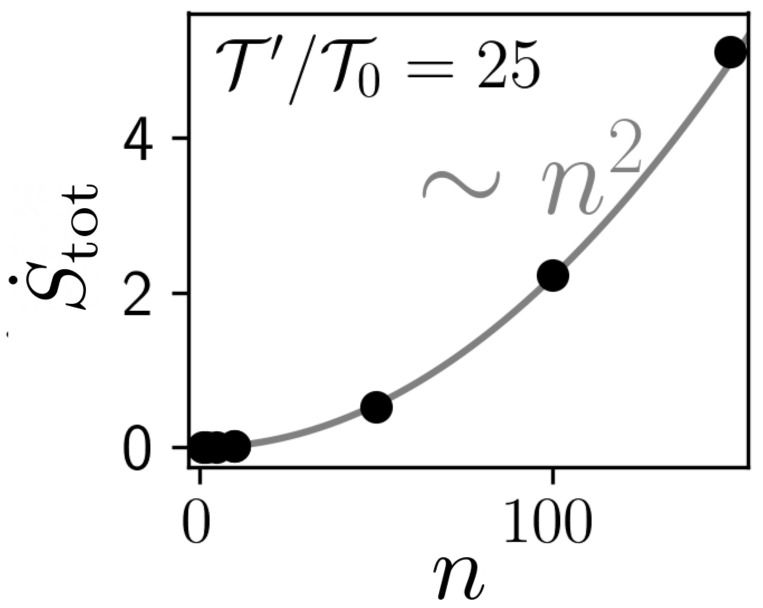
Entropy production rate in a nonlinear system with potential $V=V_{0}\left(x_{0}^{4}-2x_{0}^{2}\right)$ for different *n*; with τ=4, k=0.1, $V_{0}=1$, $T^{\prime }=1$, $T_{0}=0.04$, $k_{B}=1$. The quadratic fit is a guide to the eye. The data points stem from Brownian dynamics simulations of steady-state trajectories of Equation (5) with temporal discretization $\Delta t=10^{-4}$, and averages over a minimum of N=1000 runs with different random number seeds.

Now we discuss the dependency on *n*. The left panels of Figure 8 show that, in sharp contrast to the ${\dot{Q}}_{0}$ saturating at large *n*, the total EP generally increases with *n*, specifically we observe a *quadratic increase*$∼n^{2}$. This observation is robust against the details of the system, like *a*, *k*, τ. In fact, it even holds for *nonlinear* systems, as exemplarily shown in Figure 9 for a bistable system. We further note that the $n^{2}$ dependence persists within in the aforementioned alternative interpretation (see Section 2.4), when we scale the heat baths of $X_{j>0}$ with *n*, i.e., $T^{\prime }=nT\prime \prime$ (not shown here). The observed $n^{2}$ dependency implies a divergent EP. Taking the perspective that the $X_{j>0}$ model a memory device, where each memory cell contributes to EP, a divergent total EP is indeed expected due to the infinite system size. From an information-theoretical perspective, it is indeed also not surprising, as for n→∞ the controller stores a full stochastic trajectory, which has, due to the white noise, infinitely many jumps at each time interval, giving it an infinite information content.

The total EP also diverges if $T^{\prime }$ goes to zero, corresponding to the cases of vanishing controller errors. Thus, an infinite precision has an infinite cost. This is in accordance with the result from [75], whose limit of “precise and infinitely fast” control corresponds in our time-continuous model to the limit $T^{\prime }\to 0$. (Mathematically, the divergence of the entropy production rate as $T^{\prime }\to 0$, can simply be seen as a consequence of the definition ${\dot{S}}_{\mathrm{tot}}\propto {\dot{Q}}_{j}/T^{\prime }$, where one should note that the heat fluxes themselves do not vanish in this limit).

**Figure A1:**
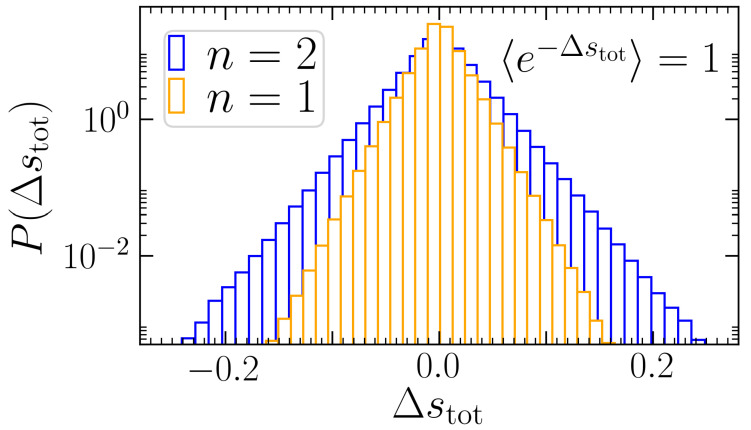
Distributions of the total EP (Equation (25)) for n=1,2 in a stable, linear system, obtained by Brownian dynamics simulations. The linear decays in this logarithmic plots indicate exponential tails. As expected, both distributions fulfill the integral fluctuation theorem $\langle e^{-\Delta s_{\mathrm{tot}}}\rangle =1$. To calculate the distributions, $>5\cdot 10^{6}$ steady-state trajectories of length $10^{4}$ were generated. All parameters and $k_{B}$ are set to unity.

Lastly, let us briefly comment on the total EP fluctuations (discussed in Appendix E). The characteristics of the entropy distributions are found to be very similar for exponential vs. non-monotonic delay distributions (specifically, Figure A1 shows n=1,2). In both cases, they exhibit exponentially decaying tails and fulfilling an integral fluctuation theorem.

## 6. Conclusions

In this work, we have studied single-particle stochastic systems under the impact of a linear delay force. We have focused on the question how the distribution of the delay affects the stability and entropy production of the stochastic system.

To this end, we have considered a one-variable Langevin equation for $X_{0}$ with white and colored noise, conservative forces stemming from an external potential, and a linear delay force that involves a Gamma-distributed delay. Gamma-distributions turn out to be particularly suitable to study these questions, because they are versatile and moreover allow for a finite representation in the form of a set of n+1 Markovian first-order differential equation (involving *n* “auxiliary variables”). This set of equations resembles a unidirectionally coupled ring network of length n+1 [41]. We note that for a general functional form of the delay distribution, a finite-dimensional representation is not guaranteed. By exploiting the Markovian representation of the dynamics, we could find analytical results for ensemble-averaged quantities in the long-time limit, where the non-Markovian process reaches a steady state.

We have found that the distribution of the delay has a tremendous impact on the linear stability, even if mean delay time and weight of delay distribution remain unchanged. Interestingly, the regime of parameter where the system is stable is smallest in the case of a delta-distributed delay, while the exponentially decaying delay represents the type of delay with the largest stability region. Another remarkable observation is that for delay distributions with a peak of nonzero width around the mean delay time, we observe a reentrant behavior by varying the mean delay time. In particular, upon increasing the mean delay time, a system may become unstable, while an even further increase of delay time stabilizes the system again. This reentrant behavior is however not observed for delta-distributed or exponentially decaying delay. While in this paper, we have discussed the linear stability of *fixed points* of (nonlinear) time-delay processes, it would further be very interesting, as a next step, to systematically consider the stability regions of different dynamical regimes of nonlinear delay systems (e.g., for different *n*). For example, nonlinear delay systems may exhibit bifurcations where stable periodic orbits emerge, associated with rich dynamical phenomena such as coherence resonance and stochastic resonance [43,58]. In fact, our preliminary numerical studies show that the coherence resonance regime that we have described in [43] for n→∞ also exists for finite values of *n*.

Studying the heat flow of $X_{0}$ in the stable parameter regions, we found that for all types of delay distribution, the mean steady-state heat rate can be positive or *negative*. Negative heat flow implies that the delay force continuously cools down the heat bath by extracting energy from it and thereby reduces the medium entropy production. This points out the fundamentally different character of a delay force and a Markovian drive, which can never on average extract energy as stated by the second law. We have shown that the exponentially decaying delay yields the largest magnitude of heat flow, i.e., extracts the most energy from the heat bath.

Last, we have considered the total entropy production of all variables in the Markovian representation of the process. This entropy production is only meaningful if we interpret the “auxiliary variables” as actual physical processes. However, this consideration offered an interesting new perspective on the feedback cooling. First, this phenomenon can be traced back to the involved non-reciprocal couplings [57]. Second, it is not due to (hidden) temperature gradients. Specifically, one might have argued that a “colder feedback controller” is expected to extract energy from a system it is coupled to. However, here we have clearly shown that also an isothermal “controller” may extract energy. We found that the total entropy production increases quadratically with *n*, implying that the controller with n=1, which yields exponentially decaying delay, has the lowest entropic cost. Taken together, the exponentially decaying delay force is the one with the largest linear stability region, which may extract the most energy from the bath, and has, at the same time, the lowest entropic cost, making it the most “robust” and “efficient” one.

The special case of discrete delay with white noise emerges as the limit n→∞ in our approach. We find that the heat exchange of the system of interest, $X_{0}$, approaches a known result from the literature [43]. On the contrary, the total entropy production diverges, suggesting that the cost of storing an infinite amount of information is unbounded.

While we have here mostly looked at linear systems, our numerical investigations demonstrate that the main effects can also be found in nonlinear systems.

A particular interesting application of our results concerns the approximation of discrete delay processes by finite numbers of *n*. Indeed, if a non-Markovian process with a single delay time of type (Equation (A16)) is the original process, one may consider the *n*-dimensional Markovian process with small numbers of *n* as an approximation to the latter (which becomes exact as n→∞). One generally expects that, the smaller *n*, the “worse” this approximation. Our results confirm this expectation. However, they moreover show that the approximation can be improved upon setting $T^{\prime }=T_{0}$. Furthermore, Figure 7c,d demonstrates that this approximation is actually already surprisingly good, even for n=1, in terms of heat flow (and, thus, average applied work by the feedback). This is somewhat surprising, given that heat and work are “sensitive” to dynamical aspects, as they depend on two-time correlation functions. Our results thus demonstrate a powerful way to approximate the discrete delay case by a very low-dimensional representation.

As a physical application for our theoretical considerations, we have here considered a colloidal particle under time-delayed feedback control, which is, in principle, experimentally realizable with optical feedback. More generally, our results carry over to other real-world systems, describable by delayed Langevin equations. Possible applications include the dynamics (and associated thermodynamics) of tracer particles in nonequilibrium viscoelastic environments [76], active environments [77], or granular media [78]. It will further be interesting to think about the physical implications of the negative heat flow reported here in other systems.

By generalizing the coupling matrix in the Markovian representation, one could use the approach presented here to further analyze more complicated delay distributions (e.g., multi-peaked ones, oscillatory delay), broadening the range of applications. We have undertaken first steps in this direction in [79], where we studied correlation functions and the nonequilibrium dynamics of systems similar to the one considered here, with up to two auxiliary variables. Further, several conclusions are indeed expected to carry over to other types of (non-Gamma) delay distributions. Indeed we have numerically observed that the $X_{0}$-dynamics essentially remains the same upon moderate variations of the delay distribution. An exception represent delay distributions that are not “short-ranged", like algebraically decaying delay distributions. These are, moreover known to yield anomalous diffusion, which might also result in interesting thermodynamic behavior and non-trivial stability bounds, representing a worthwhile perspective of future research.

Lastly, various interesting open questions concerning the thermodynamic analysis remain. For example, we have here only considered the EP of the Markovian supersystem, but we have not discussed irreversibility measures based on the non-Markovian dynamics of $X_{0}$ alone. Thermodynamic notions of systems with discrete delay were indeed recently also discussed by Rosinberg, Munakata and Tarjus [42,50,73]. They introduced such a framework of irreversibility measures based solely on the non-Markovian dynamics of $X_{0}$, focusing on the case of underdamped motion. In this context, one may for example investigate if, upon marginalization of the total entropy production calculated here, an effective irreversibility measure for the delay process, similar to [80], can be constructed.

Next, while we have so far mostly focused on the mean values, one could further explore the distributions. The mean value of the heat flow considered here is equivalent to the mean work performed on the system. For Markovian systems, the fluctuations of the work are known to fulfill useful fluctuation theorems such as the Jarzynski relation. It would be interesting to discuss generalization of such work fluctuations theorems to non-Markovian systems, taking the approach presented here as a possible starting point. For non-Makovian systems that fulfill FDR, such generalizations have indeed been carried out successfully in the past: see [81,82]. Similarly, this approach might also be useful to investigate thermodynamic uncertainty [83,84] relations in non-Markovian systems [85,86].

## Data Availability

Not Applicable.

## Abbreviations

The following abbreviations are used in this manuscript:

LE	Langevin equation
FDR	Fluctuation-dissipation relation
ODE	Ordinary differential equation
DDE	Delay differential equation
EP	Entropy production

## Appendix A. Memory Kernel and Noise Correlations

We here derive the delay distribution (Equation (3)) and the correlation of the colored noise (Equation (6)). To this end, we solve the equations for the “auxiliary variables” $X_{j\in \{1,2,...,n\}}$ given in Equation (5) in frequency space, making use of their linearity (we want to emphasize that their linearity is irrespective of the question whether the equation of $X_{0}$ is linear or not, e.g., $-V^{\prime }\left(X_{0}\right)$ might be nonlinear). First, we apply the Laplace transformation $L\left[X_{j}\left(t\right)\right]\left(s\right)=\int_{0}^{\infty }X_{j}\left(t\right)e^{-st}ds$ and obtain for each j∈{1,2,...,n}

(A1) $$s{\hat{X}}_{j}\left(s\right)=\left(n/\tau \right)\left[{\hat{X}}_{j-1}\left(s\right)-{\hat{X}}_{j}\left(s\right)\right]+\;{\hat{\xi }}_{j}\left(s\right)\Rightarrow {\hat{X}}_{j}\left(s\right)=\frac{\left(n/\tau \right){\hat{X}}_{j-1}\left(s\right)+{\hat{\xi }}_{j}\left(s\right)}{s+(n/\tau )}$$

and by iteratively substituting the solutions (Equation (A1)),

(A2) $${\hat{X}}_{n}=\frac{{\left(n/\tau \right)}^{n}}{{\left[s+\left(n/\tau \right)\right]}^{n}}{\hat{X}}_{0}+\sum_{j=1}^{n}\frac{{\left(n/\tau \right)}^{j-1}\;{\hat{\xi }}_{n-j+1}}{{\left[s+\left(n/\tau \right)\right]}^{j}}.$$

Now we transform back to the real space via inverse Laplace transformation. In Equation (A2) we identify the Laplace-transform of the Gamma-distribution $L\left[K_{j}\left(t\right)\right]\left(s\right)=\frac{{\left(n/\tau \right)}^{j}}{{\left[s+\left(n/\tau \right)\right]}^{j}}$ with the Gamma-distributed kernels $K_{j}\left(t\right)=\frac{n^{j}}{\tau^{j}\left(j-1\right)!}\;t^{j-1}e^{-nt/\tau }$. We further make use of the convolution theorem as well as the linearity of the Laplace transformation, and find

(A3) $$X_{n}\left(t\right)=\int_{0}^{t}K_{n}\left(t-t^{\prime }\right)X_{0}\left(t^{\prime }\right)\;dt^{\prime }+\nu_{n}\left(t\right)$$

with the Gaussian colored noise

(A4) $$\nu_{n}\left(t\right)=\sum_{j=1}^{n}\int_{0}^{t}\frac{\tau }{n}K_{j}\left(t-t^{\prime }\right)\;\xi_{n-j+1}\left(t^{\prime }\right)\;dt^{\prime }.$$

Due to the coupling between $X_{n}$ and $X_{0}$ in Equation (5), this yields in the dynamical Equation of $X_{0}$ (2) the colored noise $\nu =k\nu_{n}$ and the delay distribution $K=kK_{n}$ (Equation (3)).

The noise correlations $C_{\nu }\left(\Delta t\right)=\langle \nu \left(t\right)\nu \left(t+\Delta t\right)\rangle$ can be calculated exactly for an arbitrary Δt>0, as we will show in the following. First, we integrate out the δ-correlated noise terms, i.e.,

(A5) $$\begin{array}{cc} \frac{C_{\nu }\left(\Delta t\right)}{\psi } & =\sum_{j=1}^{n}\sum_{j=1}^{n}\int_{0}^{t}\int_{0}^{t+\Delta t}K_{j}\left(t-t\prime \right)K_{i}\left(t-t\prime \prime +\Delta t\right)\langle \xi_{n-j+1}\left(t^{\prime }\right)\xi_{n-i+1}\left(t^{\prime }\right)\rangle \;dt^{\prime }dt^{\prime \prime }\\ & =\sum_{j=1}^{n}\int_{0}^{t}K_{j}\left(t-t\prime \right)K_{j}\left(t-t\prime +\Delta t\right)\;dt^{\prime } \end{array}$$

with $\psi =2k_{B}T^{\prime }{\left(\tau /n\right)}^{2}k^{2}$. This further yields

(A6) $$\frac{C_{\nu }\left(\Delta t\right)}{\psi }=\sum_{j=1}^{n}\frac{{\left(n/\tau \right)}^{2j}}{\left(j-1\right){!}^{2}}\int_{0}^{t}{\left(t^{\prime 2}+t^{\prime }\Delta t\right)}^{j-1}e^{-n(\Delta t+2t^{\prime })/\tau }\;dt^{\prime }=e^{-n\Delta t/\tau }\sum_{j=1}^{n}\frac{\left(n/\tau \right)2^{1-2j}}{\left(j-1\right){!}^{2}}\;\left(*\right),$$

with

(A7) $$\left(*\right)=\int_{0}^{2tn/\tau }{\left(u^{2}+u\frac{2n}{\tau }\Delta t\right)}^{j-1}e^{-u}\;du.$$

In the last step we have performed the substitution $u=2t^{\prime }n/\tau$ and canceled several factors. The integral (*) can be simplified by using the binomial theorem, which yields

(A8) (*)=∑l=0j−1j−1l∫02tn/τu2(j−1−l)u2nΔtτle−udu=∑l=0j−12nΔtl(j−1)!τll!(j−l−1)!∫02tn/τu2j−l−2e−udu.

Since we focus on steady states, we now take the limit t→∞. Then, we perform the integration using $\int_{0}^{\infty }x^{p}e^{-x}dx=p!,$ which yields

(A9) $$\left(*\right)=\sum_{l=0}^{j-1}{\left(\frac{2n\Delta t}{\tau }\right)}^{l}\frac{(j-1)!(2j-l-2)!}{l!(j-l-1)!}.$$

(Note that transient dynamics could be treated similarly by using the incomplete Gamma function.) Combining Equations (A5) and (A9) yields the noise correlation given in Equation (6).

### Appendix A.1. Physical Motivation for the Dynamics of X j>0

Let us take a closer look at the physical motivation for the Markovian supersystem (5). In particular, we were inspired by feedback control of colloidal particles via optical tweezers.

In experimental setups, the delay is typically created within a computer’s memory, storing the past trajectory of length τ. In this case, the additional d.o.f. in the network (5) can be interpreted as the “memory cells” of the controller.

More specifically, imagine a memory device with a shift register type of architecture, as sketched in Figure 3. At every instant in time, the information stored in each cell $X_{j}$ is shifted one step further from *j* to j+1, while the new information (gathered by a measurement operation) is stored in the first cell $X_{1}$. Thus $X_{n}$ stores the oldest information and is used to perform the feedback control force $kX_{n}$. Mathematically, the updating operation can be represented in discretized form as

(A10) $$\Delta X_{j}=\frac{\Delta t}{\tau /n}\left[X_{j-1}-X_{j}\right]+\epsilon_{j},$$

where $\epsilon_{j}$ is a random number accounting for errors, e.g., from imperfect measurement, rounding operations, or a finite temperature of the device. For the sake of simplicity, we assume that the magnitude of error is identical in each memory cell. As indicated in Equation (A10), the updates of the memory cells are scaled with τ/n to enable storage of a trajectory of length τ in *n* cells. Since we aim to model time-*continuous* feedback loops, we then assume that the updates are performed (infinitely) fast, that is, Δt→0, yielding the dynamical equations of $X_{j}$ in Equation (5). As discussed in Section 2.4, there are two rather obvious ways to scale the amplitudes of the noise terms $\epsilon_{j}$ in Equation (A10). First, if the noise terms stem from a finite temperature of the controller, it seems appropriate to scale them with equal strength $∼T^{\prime }$, independent of the number of *n*. This is the main interpretation considered in this paper. Alternatively, if the $\epsilon_{j}$ are assumed to stem from imperfect measurement, rounding errors, an erroneous information transfer or computation within each cell and at each time step, it may be more realistic to also scale the $\epsilon_{j}$-terms with $\sqrt{n/\tau }$ (recall that the dimension of a noise terms in Langevin equations is “$\sqrt{\mathrm{time}}$”). This then results in Gaussian white noise terms $\xi_{j>0}$ with $\langle \xi_{j}\xi_{i>0}\rangle =2k_{B}T\prime \prime n/\tau$ in Equation (5). In turn, this yields a colored noise with *n*-independent weight, see Section 2.4.

As suggested in [87], such a memory device could be realized by *n* colloidal particles at positions $X_{j}$, each trapped in a harmonic trap of stiffness n/τ centered around the position of the proceeding colloid, $X_{j-1}$. This idea is, of course, only a thought experiment. Still, it is useful to illustrate how a delay distribution concentrated around a specific instant in time of the past (the delay time), can result from coupled exponential relaxation processes. It further demonstrates that an implementation of (non-monotonic) memory with physical d.o.f. is inevitably associated with thermodynamic cost and dissipation, which we discuss in Section 4.

## Appendix B. Analytical Solutions for Correlations

Here we sketch how analytical expressions for the correlations $\langle X_{i}\left(t\right)X_{j}\left(t\right)\rangle$ can be obtained for arbitrary system sizes *n*. To this end, we transform Equation (5) via the Fourier transformation ${\tilde{X}}_{j}\left(s\right)=\int_{-\infty }^{\infty }X_{j}\left(t\right)e^{-i\omega t}dt$, which readily yields

(A11) $$i\omega \underline{\tilde{X}}\left(\omega \right)=A\underline{\tilde{X}}\left(\omega \right)+\underline{\tilde{\xi }}\left(\omega \right)\Rightarrow \underline{\tilde{X}}\left(\omega \right)=\underset{=\underline{\underline{\tilde{\lambda }}}\left(\omega \right)}{\underset{︸}{{\left(i\omega 1-A\right)}^{-1}}}\;\underline{\tilde{\xi }}\left(\omega \right),$$

with the unity matrix 1 and the Green’s function in Fourier-space $\underline{\underline{\tilde{\lambda }}}\left(\omega \right)$, determined by the inverse of the topology matrix A. Using the well-known relationship between spatial correlations and the Green’s function from linear response theory [88]

(A12) $$C\left(\Delta t\right)=\frac{k_{B}T}{\pi }\int_{-\infty }^{\infty }\tilde{\lambda }\left(-\omega \right)\tilde{\lambda }\left(-\omega \right)\;e^{-i\omega \Delta t}d\omega ,$$

one readily finds an analytical expression

(A13) $$\langle X_{j}X_{l}\rangle =\sum_{p=0}^{n}\frac{k_{B}T_{p}}{\pi }\int_{-\infty }^{\infty }{\tilde{\lambda }}_{jp}\left(\omega \right){\tilde{\lambda }}_{lp}\left(-\omega \right)\;d\omega .$$

for arbitrary system sizes *n*, with $T_{j\neq 0}=T^{\prime }$.

While this strategy in principle yields analytical expressions for various (linear) systems (which can, e.g., be numerically integrated), explicit closed-form solutions are only available for specific cases, where the inverse Fourier transformation is known (see [38,89] for some explicit results). For example, the correlations for n=1 are given in [57,72]. We could not find general closed-from solutions for the problem with n>1.

The matrix inversion in Equation (A11) is indeed possible up to very large system sizes, as the coupling matrix A is sparse. To evaluate the integrals in Equation (A13), the residue theorem can be used. However, this requires finding the roots of a polynomial of order n+1. Using computer algebra systems, this can be done reasonably fast up to about n=10. We also note that we could find for the case $T^{\prime }=0$ solutions up to $n∼10^{4}$ in this way.

## Appendix C. Path Probabilities with Onsager–Machlup Action

The path probability P conditioned on the starting point $\underline{X}\left(t_{s}\right)={\underline{x}}_{s}$, is given by the exponential of the Onsager–Machlup action [70,90]. The later readily follow from the LEs (Equation (5)), and read

(A14) $$P\left[\underline{X}|{\underline{x}}_{s}\right]\propto \exp\;\left[-\sum_{j=0}^{n}\int_{t_{s}}^{t_{f}}\frac{{\left\{\gamma_{j}{\dot{X}}_{j}\left(t^{\prime }\right)-F_{j}\left[\underline{X}\left(t^{\prime }\right)\right]\right\}}^{2}}{4k_{B}T_{j}\gamma_{j}}dt^{\prime }\right],$$

and

(A15) $$\hat{P}\left[\hat{\underline{X}}|{\underline{x}}_{f}\right]\propto \;\exp\;\left[-\sum_{j=0}^{n}\int_{t_{s}}^{t_{f}}\frac{{\left\{-\gamma_{j}{\dot{X}}_{j}\left(t^{\prime }\right)-F_{j}\left[\underline{X}\left(t^{\prime }\right)\right]\right\}}^{2}}{4k_{B}T_{j}\gamma_{j}}dt^{\prime }\right],$$

with $F_{j>0}=-n/\tau X_{j}+n/\tau X_{j-1}$, $\gamma_{j>0}=1$, $T_{j>0}=T^{\prime }$, and $F_{0}=-a_{}X_{0}+kX_{n}.$$\hat{P}$ is the corresponding backwards path probability of the corresponding backward process. We here assume that $X_{j>0}$ are even under time-reversal, like positions, angles, or orientations, which is more suitable when $X_{j>0}$ model memory cells of a feedback-controller (odd parity would imply information storage in particle velocities).

## Appendix D. Limit of the Heat Flow

The limit of the heat flow for n→∞ corresponds to the heat flow in the system with discrete delay and white noise, which reads [43]

(A16) $${\dot{Q}}_{0}=k^{2}\langle X_{n}^{2}\rangle -ka_{}\langle X_{0}\left(t\right)X_{0}\left(t-\tau \right)\rangle =a_{}+k\delta_{\tau ,0}-\frac{1-k\sinh\left[\tau \sqrt{a_{}^{2}-k^{2}}\right]/\sqrt{a_{}^{2}-k^{2}}}{\left(a_{}-k\cosh\left[\tau \sqrt{a_{}^{2}-k^{2}}\right]\right)/\left(a_{}^{2}-k^{2}\right)},$$

with the Kronecker delta $\delta_{\tau ,0}$ which is 1 at τ=0 and zero elsewhere.

## Appendix E. Entropy Distributions for Different Types of Delay Distributions

While we mainly focus on the ensemble-averages of heat and entropy in the main text, we here briefly discuss the distributions of the total entropy production. Figure A1 displays numerically obtained distributions of the total EP fluctuations in a linear system with delay, and two different delay distributions. Remarkably, the entropy distributions look very similar for n=1 and 2, despite the distinct characteristics of the delay distributions in both cases (exponential decay vs. non-monotonic). In particular, both distributions have exponentially decaying tails. As expected, both cases further fulfill an integral fluctuation theorem $\langle e^{-\Delta S_{\mathrm{tot}}}\rangle =1$.
